# State-Specific Prevalence of Current Cigarette Smoking and Smokeless Tobacco Use Among Adults Aged ≥18 Years — United States, 2011–2013

**Published:** 2015-05-22

**Authors:** Kimberly Nguyen, LaTisha Marshall, Sean Hu, Linda Neff

**Affiliations:** 1 Office on Smoking and Health, National Center for Chronic Disease Prevention and Health Promotion, CDC

Cigarette smoking and the use of smokeless tobacco both cause substantial morbidity and premature mortality (1,2). The concurrent use of these products might increase dependence and the risk for tobacco-related disease and death (1,2). State-specific estimates of prevalence and relative percent change in current cigarette smoking, smokeless tobacco use, and concurrent cigarette smoking and smokeless tobacco use among U.S. adults during 2011–2013, developed using data from the Behavioral Risk Factor Surveillance System (BRFSS), indicate statistically significant (p<0.05) changes for all three behaviors. From 2011 to 2013, there was a statistically significant decline in current cigarette smoking prevalence overall and in 26 states. During the same period, use of smokeless tobacco significantly increased in four states: Louisiana, Montana, South Carolina, and West Virginia; significant declines were observed in two states: Ohio and Tennessee. In addition, the use of smokeless tobacco among cigarette smokers (concurrent use) significantly increased in five states (Delaware, Idaho, Nevada, New Mexico, and West Virginia). Although annual decreases in overall cigarette smoking among adults in the United States have occurred in recent years (2), there is much variability in prevalence of cigarette smoking, smokeless tobacco, and concurrent use across states. In 2013, the prevalence ranged from 10.3% (Utah) to 27.3% (West Virginia) for cigarette smoking; 1.5% (District of Columbia and Massachusetts) to 9.4% (West Virginia) for smokeless tobacco; and 3.1% (Vermont) to 13.5% (Idaho) for concurrent use. These findings highlight the importance of sustained comprehensive state tobacco-control programs funded at CDC-recommended levels, which can accelerate progress toward reducing tobacco-related disease and deaths by promoting evidence-based population-level interventions. These interventions include increasing the price of tobacco products, implementing comprehensive smoke-free laws, restricting tobacco advertising and promotion, controlling access to tobacco products, and promoting cessation assistance for smokers to quit, as well as continuing and implementing mass media campaigns that contain graphic anti-smoking ads, such as the Tips from Former Smokers (TIPS) campaign (3).

BRFSS is a state-based telephone survey of noninstitutionalized U.S. adults aged ≥18 years; in 2011, the survey began using data obtained from both landline and cell phone samples. The median state response rates during 2011–2013 were 49.7% (2011), 45.2% (2012), and 45.9% (2013). The survey assessed prevalence of current cigarette smoking* and current smokeless tobacco use.† State-specific point prevalence for current cigarette smoking and current smokeless tobacco use was calculated for all 50 U.S. States and the District of Columbia. In addition, the prevalence of smokeless tobacco use among cigarette smokers was calculated to determine an estimate of concurrent use of both products. Estimates were weighted to adjust for differences in the probability of selection and nonresponse, and 95% confidence intervals were computed.§ The relative percent change (RPC) in prevalence during 2011–2013 was also calculated.¶ Logistic regression was used to assess trends over time adjusting for sex, age, and race/ethnicity; the Wald test was used to determine statistical significance (p<0.05). The analysis was restricted to 2011–2013 because of a change in the weighting methodology and the addition of cell phone samples beginning in 2011.**

Current cigarette smoking ranged from 11.8% (Utah) to 29.0% (Kentucky) in 2011 and from 10.3% (Utah) to 27.3% (West Virginia) in 2013 (Table 1). During 2011–2013, current cigarette smoking declined significantly in 26 states: Arizona, Florida, Georgia, Hawaii, Illinois, Indiana, Kansas, Kentucky, Maine, Maryland, Michigan, Missouri, Montana, Nebraska, Nevada, New Hampshire, New Mexico, Oklahoma, Oregon, Rhode Island, South Dakota, Texas, Utah, Vermont, Wisconsin, and Wyoming (Figure 1). No significant changes were observed in any other states.

Current smokeless tobacco use ranged from 1.4% (California and Rhode Island) to 9.8% (Wyoming) in 2011 and from 1.5% (District of Columbia and Massachusetts) to 9.4% (West Virginia) in 2013 (Table 1). Increases (RPCs) were observed in four states: Louisiana (26.7%), Montana (12.7%), South Carolina (22.2%), and West Virginia (25.3%), while declines were observed in smokeless tobacco use in two states: Ohio (−16.0%) and Tennessee (−25.0%) (Figure 2).

The prevalence of concurrent use of cigarettes and smokeless tobacco ranged from 2.0% (Nevada) to 12.5% (Utah) in 2011, and from 3.1% (Vermont) to 13.5% (Idaho) in 2013 (Table 2). Significant increases (RPCs) in concurrent use were observed in five states: Delaware (100.0%), Idaho (57.0%), Nevada (155.0%), New Mexico (25.4%), and West Virginia (31.3%); no significant changes were observed in other states.

## Discussion

States vary widely in prevalence of cigarette smoking, smokeless tobacco, and concurrent use of both products. The overall prevalence of current cigarette smoking declined significantly in approximately half of U.S. states during 2011–2013; however, there has been relatively little change in the prevalence of current smokeless tobacco or concurrent use of cigarettes and smokeless tobacco in most states during this period, with prevalence increasing in some states. The use of more than one tobacco product is concerning because persons aged ≥18 years who use both cigarettes and smokeless tobacco have higher levels of nicotine dependence and are less likely to report planning to quit than those who exclusively smoke cigarettes (4). Although multiple components of tobacco control prevention and policy have had an effect on reducing cigarette smoking overall and within most states (2), the varied prevalence and increases in smokeless tobacco use in some states highlights the importance of targeted population-based interventions focused on reducing the use of all tobacco products.

What is already known on this topic?

Cigarette smoking and the use of smokeless tobacco both cause substantial morbidity and premature mortality. The concurrent use of these products might increase dependence and the risk for tobacco-related disease and death.

What is added by this report?

During 2011–2013, cigarette smoking prevalence declined significantly in 26 states. However, smokeless tobacco use declined in only two states (Ohio and Tennessee) and increased in four states (Louisiana, Montana, South Carolina, and West Virginia). A significant increase in concurrent use of cigarettes and smokeless tobacco was observed in five states (Delaware, Idaho, Nevada, New Mexico, and West Virginia).

What are the implications for public health practice?

The findings in this report underscore the importance of implementing proven interventions for reducing the use of all tobacco products. Full implementation of comprehensive tobacco control programs at CDC-recommended funding levels, in conjunction with the Food and Drug Administration regulation of tobacco products, could reduce tobacco use and change social norms regarding the acceptability of tobacco use in the United States.

Although a statistically significant change in cigarette smoking prevalence occurred in 26 states, no change occurred in 24 states and the District of Columbia. In addition, smokeless tobacco use prevalence decreased in only two states (Ohio and Tennessee), while prevalence increased in four states (Louisiana, Montana, South Carolina, and West Virginia). Smokeless tobacco use among current cigarette smokers increased by more than 50% in one state (Idaho) and more than doubled in two states (Delaware and Nevada). These increases could be attributable to increases in marketing of smokeless tobacco, the misperception that smokeless tobacco is a safe alternative to cigarettes, and the lower price of smokeless tobacco products relative to cigarettes in most states (1,4). In addition, the tobacco industry has marketed smokeless tobacco as an alternative in areas where smoking is otherwise prohibited (5). As of January 2015, a total of 26 states (not necessarily those that saw smoking decreases) and the District of Columbia have implemented comprehensive smoke-free laws that prohibit smoking in all indoor areas of worksites, restaurants, and bars (6).

This report provides the most recent state-based estimates of current cigarette smoking and smokeless tobacco use for all 50 states and the District of Columbia. The estimates are produced using new weighting methods (e.g., raking) in BRFSS that include both landline and cell phone-only households to increase generalizability.§ However, this study is subject to at least three limitations. First, the estimates for tobacco use were self-reported. Although studies of self-reported smoking have been shown to yield lower prevalence estimates than studies using serum cotinine (7), a metabolite of nicotine, underreporting likely did not have a large effect on the trends described in this report (8). Second, the BRFSS sampling frame does not include adults without telephone service; however, their exclusion would not be expected to introduce any major bias because only 1.8% of U.S. adults reported having no telephone service in 2011 (9). Finally, the median state response rates ranged from 49.7% (2011), 45.2% (2012), and 45.9% (2013). Lower response rates can increase the potential for bias; however, overall estimates from state-aggregated BRFSS data are comparable to smoking estimates from national surveys with higher response rates (10).

Although overall cigarette smoking prevalence has declined significantly in recent years in many states, the overall use of smokeless tobacco and concurrent cigarette and smokeless tobacco has remained unchanged in most states and increased in some states. The findings in this report underscore the importance of implementing proven interventions for reducing the use of all tobacco products, including increasing the price of tobacco products, implementing comprehensive smoke-free policies and mass media campaigns, restricting tobacco advertising and promotion, controlling access to tobacco products, promoting cessation assistance for tobacco users to quit, and federal regulation of the manufacturing, distribution, and marketing of tobacco products (3). Evidence-based, statewide tobacco-control programs that are comprehensive, sustained, and accountable have been shown to reduce smoking rates, as well as tobacco-related diseases and deaths (3). However, during 2015, despite combined revenue of more than $25 billion from settlement payments and tobacco taxes for all states, states will spend only $490.4 million (1.9%) on comprehensive tobacco-control programs,†† representing <15% of the CDC-recommended level of funding for all states combined (3). Full implementation of comprehensive tobacco control programs at CDC-recommended funding levels, in conjunction with the Food and Drug Administration regulation of tobacco products, could further reduce all forms of tobacco use (3).

## Figures and Tables

**FIGURE 1:**
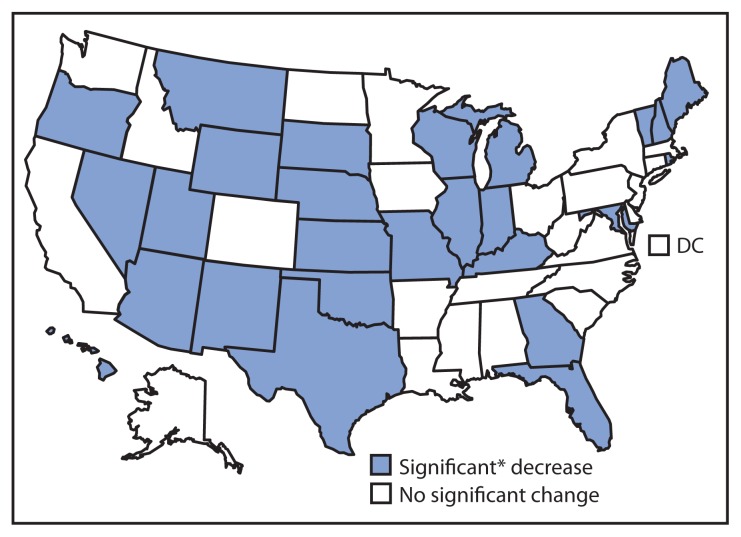
Change in percentage of current cigarette smoking among adults — Behavioral Risk Factor Surveillance System, United States, 2011–2013 * Significant = p<0.05.

**FIGURE 2:**
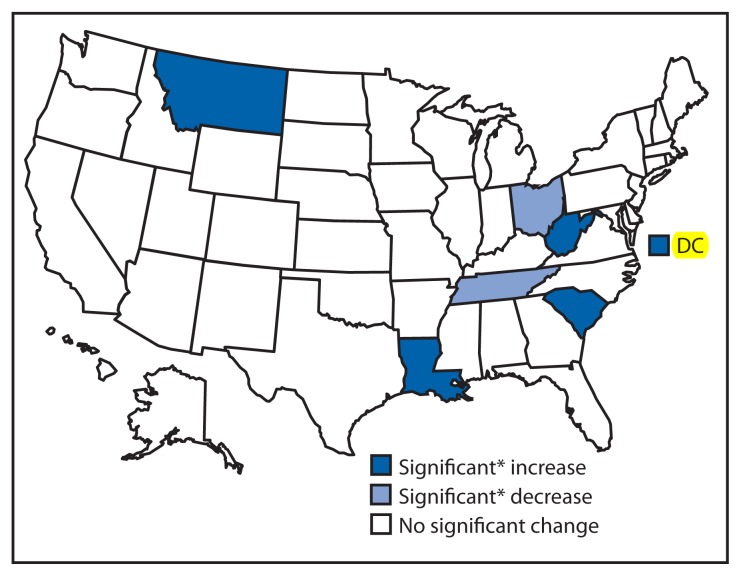
Change in percentage of current smokeless tobacco use among adults — Behavioral Risk Factor Surveillance System, United States, 2011–2013 * Significant = p<0.05.

**TABLE 1:** State-specific prevalence of current cigarette smoking* and current smokeless tobacco use† among adults aged ≥18 years, by state/territory — Behavioral Risk Factor Surveillance System, United States, 2011–2013

State/Territory	Cigarette smoking							Smokeless tobacco						
	
	2011		2012		2013		RPC§	2011		2012		2013		RPC
					
	%	(95% CI)	%	(95% CI)	%	(95% CI)		%	(95% CI)	%	(95% CI)	%	(95% CI)	
Alabama	24.3	(22.9–25.8)	23.8	(22.4–25.2)	21.5	(19.9–23.1)	11.5	6.5	(5.7–7.4)	5.8	(5.0–6.6)	6.1	(5.2–7.1)	−6.2
Alaska	22.9	(21.0–25.0)	20.5	(18.9–22.3)	22.6	(20.8–24.4)	−1.3	5.9	(4.9–7.1)	6.1	(5.2–7.1)	6.8	(5.8–7.8)	15.3
Arizona	19.3	(17.2–21.3)	17.1	(15.6–18.6)	16.3	(14.4–18.4)	−15.5¶	3.1	(2.4–4.0)	3.1	(2.5–3.9)	3.2	(2.4–4.2)	3.2
Arkansas	27.0	(24.8–29.2)	25.0	(23.4–26.8)	25.9	(24.1–27.8)	−4.1	7.1	(5.8–8.5)	7.1	(6.1–8.3)	6.9	(5.9–8.0)	−2.8
California	13.7	(12.9–14.4)	12.6	(11.8–13.4)	12.5	(11.7–13.5)	−8.8	1.4	(1.2–1.7)	1.3	(1.1–1.6)	1.6	(1.2–1.9)	14.3
Colorado	18.3	(17.2–19.4)	17.7	(16.8–18.7)	17.7	(16.8–18.6)	−3.3	4.5	(3.9–5.1)	4.2	(3.7–4.8)	4.2	(3.8–4.8)	−6.7
Connecticut	17.1	(15.8–18.6)	16.0	(14.9–17.2)	15.5	(14.3–16.8)	−9.4	1.5	(1.2–2.0)	1.9	(1.5–2.4)	1.8	(1.4–2.5)	20.0
Delaware	21.8	(19.9–23.6)	19.7	(18.2–21.3)	19.6	(18.0–21.2)	−10.1	2.2	(1.6–2.9)	2.5	(1.9–3.3)	2.2	(1.7–2.9)	0.0
District of Columbia	20.8	(18.8–22.9)	19.6	(17.4–22.0)	18.8	(16.9–20.9)	−9.6	1.5	(1.0–2.2)	1.6	(0.9–2.8)	1.5	(0.9–2.3)	0.0
Florida	19.3	(18.2–20.5)	17.7	(16.3–19.2)	16.8	(16.0–17.8)	−13.0¶	3.0	(2.5–3.6)	3.2	(2.6–3.9)	2.6	(2.3–3.0)	−13.3
Georgia	21.2	(19.9–22.6)	20.4	(18.9–22.0)	18.8	(17.6–20.1)	−11.3¶	4.4	(3.8–5.1)	4.1	(3.4–4.9)	5.0	(4.3–5.8)	13.6
Hawaii	16.8	(15.5–18.3)	14.6	(13.3–15.9)	13.3	(12.2–14.5)	−20.8¶	1.9	(1.4–2.4)	2.0	(1.5–2.6)	1.7	(1.3–2.1)	−10.5
Idaho	17.2	(15.6–18.9)	16.4	(14.7–18.3)	17.2	(15.7–18.8)	0.0	4.8	(4.0–5.8)	4.9	(3.9–6.1)	5.7	(4.7–6.8)	18.8
Illinois	20.9	(19.2–22.7)	18.6	(17.0–20.3)	18.0	(16.6–19.6)	−13.9¶	3.4	(2.7–4.2)	2.5	(1.8–3.3)	2.6	(2.0–3.3)	−23.5
Indiana	25.6	(24.3–27.0)	24.0	(22.8–25.2)	21.9	(20.8–23.1)	−14.5¶	5.0	(4.3–5.8)	4.0	(3.5–4.7)	4.9	(4.3–5.5)	−2.0
Iowa	20.4	(19.1–21.6)	18.1	(17.0–19.3)	19.5	(18.3–20.7)	−4.4	4.2	(3.6–4.9)	4.3	(3.7–5.1)	4.9	(4.2–5.7)	16.7
Kansas	22.0	(21.2–22.8)	19.4	(18.4–20.4)	20.0	(19.3–20.7)	−9.1¶	5.3	(4.9–5.8)	5.5	(4.9–6.2)	5.5	(5.1–5.9)	3.8
Kentucky	29.0	(27.5–30.5)	28.3	(26.9–29.7)	26.5	(25.1–27.8)	−8.6¶	6.8	(6.0–7.6)	6.1	(5.4–6.9)	7.0	(6.3–7.9)	2.9
Louisiana	25.7	(24.3–27.2)	24.8	(23.2–26.3)	23.5	(21.5–25.6)	−8.6	4.5	(3.8–5.2)	5.6	(4.8–6.5)	5.7	(4.7–6.9)	26.7¶
Maine	22.8	(21.7–23.9)	20.3	(19.2–21.4)	20.2	(19.0–21.5)	−11.4¶	2.8	(2.4–3.3)	2.2	(1.9–2.7)	2.0	(1.6–2.6)	−28.6
Maryland	19.1	(17.8–20.5)	16.2	(15.0–17.4)	16.4	(15.3–17.5)	−14.1¶	2.1	(1.7–2.7)	2.0	(1.5–2.5)	2.5	(2.0–3.1)	19.0
Massachusetts	18.2	(17.3–19.2)	16.4	(15.6–17.2)	16.6	(15.6–17.7)	−8.8	1.7	(1.4–2.1)	1.3	(1.1–1.6)	1.5	(1.2–1.9)	−11.8
Michigan	23.3	(22.0–24.6)	23.3	(22.1–24.6)	21.4	(20.3–22.5)	−8.2¶	4.4	(3.8–5.1)	3.9	(3.3–4.5)	3.9	(3.5–4.5)	−11.4
Minnesota	19.1	(18.1–20.1)	18.8	(17.8–19.8)	18.0	(16.9–19.3)	−5.8	4.8	(4.3–5.4)	4.2	(3.7–4.7)	5.0	(4.3–5.8)	4.2
Mississippi	26.0	(24.6–27.4)	24.0	(22.5–25.5)	24.8	(23.3–26.4)	−4.6	8.0	(7.2–8.9)	7.5	(6.6–8.4)	8.5	(7.5–9.6)	6.3
Missouri	25.0	(23.5–26.6)	23.9	(22.4–25.5)	22.1	(20.6–23.6)	−11.6¶	5.3	(4.5–6.2)	5.1	(4.3–6.0)	5.1	(4.4–6.0)	−3.8
Montana	22.1	(20.8–23.4)	19.7	(18.6–20.9)	19.0	(17.9–20.1)	−14.0¶	7.1	(6.4–7.9)	8.0	(7.2–8.8)	8.0	(7.3–8.8)	12.7¶
Nebraska	20.0	(19.3–20.7)	19.7	(18.9–20.6)	18.5	(17.5–19.5)	−7.5¶	5.6	(5.2–6.0)	5.1	(4.6–5.6)	5.3	(4.7–5.9)	−5.4
Nevada	22.9	(21.0–25.0)	18.1	(16.6–19.8)	19.4	(17.4–21.5)	−15.3¶	2.3	(1.7–3.1)	3.7	(2.9–4.7)	3.2	(2.5–3.9)	39.1
New Hampshire	19.4	(18.0–20.9)	17.2	(15.8–18.6)	16.2	(15.0–17.6)	−16.5¶	3.0	(2.3–3.8)	2.1	(1.6–2.7)	2.7	(2.1–3.3)	−10.0
New Jersey	16.8	(15.9–17.8)	17.3	(16.4–18.3)	15.7	(14.7–16.7)	−6.5	1.6	(1.3–2.0)	1.2	(1.0–1.5)	1.7	(1.4–2.1)	6.2
New Mexico	21.5	(20.3–22.7)	19.3	(18.2–20.5)	19.1	(17.9–20.3)	−11.2¶	4.2	(3.6–4.9)	4.3	(3.7–4.9)	4.3	(3.8–5.0)	2.4
New York	18.1	(16.9–19.4)	16.2	(14.9–17.6)	16.6	(15.5–17.8)	−8.3	2.3	(1.8–2.9)	1.9	(1.5–2.4)	2.2	(1.8–2.6)	−4.3
North Carolina	21.8	(20.5–23.1)	20.9	(19.9–21.9)	20.3	(19.1–21.5)	−6.9	5.2	(4.5–5.9)	4.1	(3.6–4.6)	4.3	(3.7–5.0)	−17.3
North Dakota	21.9	(20.3–23.5)	21.2	(19.6–22.9)	21.2	(19.8–22.7)	−3.2	7.6	(6.5–8.7)	7.3	(6.2–8.5)	7.6	(6.7–8.6)	0.0
Ohio	25.1	(23.8–26.4)	23.3	(22.2–24.4)	23.4	(22.2–24.6)	−6.8	5.0	(4.3–5.7)	4.6	(4.1–5.2)	4.2	(3.7–4.8)	−16.0¶
Oklahoma	26.1	(24.7–27.6)	23.3	(22.0–24.6)	23.7	(22.4–25.0)	−9.2¶	6.9	(6.1–7.9)	6.7	(5.9–7.5)	6.3	(5.6–7.1)	−8.7
Oregon	19.7	(18.3–21.2)	17.9	(16.4–19.4)	17.3	(15.9–18.8)	−12.2¶	4.4	(3.7–5.2)	3.8	(3.1–4.8)	4.6	(3.8–5.4)	4.5
Pennsylvania	22.4	(21.3–23.6)	21.4	(20.4–22.3)	21.0	(19.9–22.0)	−6.2	4.4	(3.9–5.1)	4.3	(3.8–4.7)	4.4	(3.8–4.9)	0.0
Rhode Island	20.0	(18.6–21.5)	17.4	(16.0–18.9)	17.4	(16.1–18.8)	−13.0¶	1.4	(1.0–1.9)	1.0	(0.7–1.4)	1.9	(1.4–2.6)	35.7
South Carolina	23.1	(21.9–24.4)	22.5	(21.4–23.7)	22.0	(20.8–23.2)	−4.8	3.6	(3.1–4.2)	3.9	(3.3–4.5)	4.4	(3.8–5.1)	22.2¶
South Dakota	23.0	(21.1–25.0)	22.0	(20.5–23.5)	19.6	(18.1–21.2)	−14.8¶	6.8	(5.7–8.2)	6.4	(5.5–7.3)	6.6	(5.6–7.7)	−2.9
Tennessee	23.0	(20.7–25.5)	24.9	(23.4–26.4)	24.3	(22.6–26.1)	5.7	6.4	(5.0–8.1)	5.0	(4.3–5.9)	4.8	(3.9–5.9)	−25.0¶
Texas	19.2	(18.0–20.4)	18.2	(17.0–19.4)	15.9	(14.8–17.0)	−17.2¶	3.9	(3.4–4.5)	3.9	(3.4–0.2)	4.3	(3.7–4.9)	10.3
Utah	11.8	(11.0–12.7)	10.6	(9.8–11.4)	10.3	(9.6–11.1)	−12.7¶	3.0	(2.6–3.5)	3.0	(2.5–3.5)	2.9	(2.5–3.3)	−3.3
Vermont	19.1	(17.7–20.5)	16.5	(15.2–17.9)	16.6	(15.4–17.9)	−13.1¶	2.7	(2.1–3.3)	3.0	(2.4–3.8)	2.8	(2.2–3.5)	3.7
Virginia	20.9	(19.4–22.5)	19.0	(17.7–20.3)	19.0	(17.9–20.2)	−9.1	4.3	(3.6–5.2)	4.3	(3.7–5.2)	4.0	(2.2–3.5)	−7.0
Washington	17.5	(16.4–18.7)	17.2	(16.3–18.1)	16.1	(15.1–17.1)	−8.0	3.6	(3.0–4.2)	3.8	(3.3–4.3)	3.7	(3.4–4.6)	2.8¶
West Virginia	28.6	(27.0–30.3)	28.2	(26.7–29.7)	27.3	(25.9–28.7)	−4.5	7.5	(6.6–8.5)	8.6	(7.7–9.6)	9.4	(8.5–10.5)	25.3¶
Wisconsin	20.9	(19.2–22.7)	20.4	(18.7–22.1)	18.7	(17.2–20.3)	−10.5¶	4.0	(3.3–4.8)	4.3	(3.5–5.2)	4.3	(3.6–5.2)	7.5
Wyoming	23.0	(21.5–24.6)	21.8	(19.9–23.7)	20.6	(19.1–22.2)	−10.4¶	9.8	(8.9–10.9)	8.2	(7.0–9.6)	8.8	(7.7–10.0)	−10.2
**Median prevalence all states** **	**21.2**		**19.6**		**19.0**		−**10.4**¶	**4.4**		**4.1**		**4.3**		**2.3**

**Abbreviations:** CI = confidence interval; RPC = relative percent change (see below).

* Persons aged ≥18 years who reported having smoked ≥100 cigarettes during their lifetime and smoke every day or some days at the time of survey.

† Persons aged ≥18 years who reported currently using chewing tobacco, snuff, or snus (a small pouch of smokeless tobacco) every day or some days at the time of survey.

§ RPC was calculated by dividing the difference between the 2013 and 2011 estimates by the 2011 estimates, and expressed as a percentage.

¶ p<0.05 for trend (2011–2013) in multivariate logistic regression model adjusted for sex, age, and race/ethnicity.

** Median prevalence across all 50 U.S. states and the District of Columbia.

**TABLE 2:** Percentage of current cigarette smokers* who also currently use smokeless tobacco† among adults aged ≥18 years, by state/territory — Behavioral Risk Factor Surveillance System, United States, 2011–2013

State/Territory	2011		2012		2013		RPC§
		
	%	95% CI	%	95% CI	%	95% CI	
Alabama	8.5	(6.5–11.1)	6.1	(4.6–8.1)	7.9	(6.0–10.5)	−7.1
Alaska	7.9	(5.7.11.0)	8.6	(6.5–11.5)	8.8	(6.5–11.6)	11.4
Arizona	5.5	(3.5–8.5)	5.4	(3.6–7.9)	7.4	(4.6–11.6)	34.5
Arkansas	11.3	(8.1–15.5)	8.0	(5.9–10.8)	8.7	(6.6–11.4)	−23.0
California	4.2	(3.1–5.6)	3.3	(2.4–4.5)	3.6	(2.3–5.7)	−14.3
Colorado	7.8	(6.1–10.0)	5.3	(4.1–6.9)	7.8	(6.2–9.7)	0.0
Connecticut	2.8	(1.8–4.3)	6.6	(4.6–9.2)	3.7	(2.2–6.1)	32.1
Delaware	2.9	(1.7–5.1)	5.5	(3.5–8.6)	5.8	(3.8–8.9)	100.0¶
District of Columbia	4.6	(2.8–7.4)	4.2	(1.8–9.3)	4.6	(2.4–8.6)	0.0
Florida	5.6	(4.2–7.4)	6.2	(4.1–9.2)	5.2	(4.0–6.7)	−7.1
Georgia	7.7	(5.7–10.4)	6.5	(4.6–9.2)	7.3	(5.6–9.5)	−5.2
Hawaii	3.9	(2.5–5.9)	5.6	(3.8–8.1)	4.4	(3.0–6.5)	12.8
Idaho	8.6	(6.0–12.2)	8.2	(5.3–12.4)	13.5	(10.2–17.7)	57.0¶
Illinois	5.8	(3.8–8.8)	5.5	(3.1–9.5)	5.4	(3.7–7.8)	−6.9
Indiana	7.4	(5.8–9.6)	6.1	(4.6–7.9)	7.8	(6.3–9.6)	5.4
Iowa	7.9	(6.0–10.4)	5.1	(3.7–7.1)	7.5	(5.7–10.0)	−5.1
Kansas	8.0	(6.8–9.4)	7.0	(5.5–8.8)	8.3	(7.2–9.5)	3.8
Kentucky	8.9	(7.2–11.0)	7.4	(5.8–9.4)	10.2	(8.4–12.4)	14.6
Louisiana	6.7	(5.0–8.9)	6.9	(5.2–9.1)	6.6	(4.4–9.9)	−1.5
Maine	5.1	(3.8–6.7)	5.2	(3.8–7.0)	3.9	(2.7–5.6)	−23.5
Maryland	4.5	(2.9–6.8)	4.0	(2.6–6.1)	5.5	(3.9–7.8)	22.2
Massachusetts	4.3	(3.1–5.8)	2.1	(1.5–2.9)	4.0	(2.7–6.0)	−7.0
Michigan	9.6	(7.7–11.8)	6.7	(5.3–8.4)	8.9	(7.4–10.7)	−7.3
Minnesota	9.2	(7.5–11.2)	8.1	(6.7–9.9)	9.6	(7.6–12.1)	4.3
Mississippi	10.4	(8.4–12.7)	9.4	(7.3–12.1)	10.0	(7.9–12.5)	−3.8
Missouri	7.0	(5.1–9.5)	7.6	(5.5–10.3)	6.4	(4.7–8.5)	−8.6
Montana	10.2	(8.4–12.3)	11.6	(9.5–14.0)	12.2	(10.2–14.4)	19.6
Nebraska	9.4	(8.2–10.8)	7.9	(6.6–9.5)	9.0	(7.2–11.4)	−4.3
Nevada	2.0	(1.1–3.8)	5.1	(3.2–8.0)	5.1	(3.4–7.7)	155.0¶
New Hampshire	6.8	(4.6–10.0)	4.0	(2.3–6.9)	5.3	(3.6–7.9)	−22.1
New Jersey	3.1	(2.2–4.6)	3.0	(2.1–4.3)	4.0	(2.8–5.7)	29.0
New Mexico	7.1	(5.4–9.3)	7.4	(5.8–9.4)	8.9	(7.2–11.1)	25.4¶
New York	5.2	(3.7–7.1)	6.5	(4.8–8.9)	5.3	(4.0–7.0)	1.9
North Carolina	6.5	(4.9–8.7)	5.3	(4.2–6.7)	5.6	(4.1–7.5)	−13.8
North Dakota	11.0	(8.4–14.2)	11.0	(8.3–14.5)	11.3	(9.1–14.0)	2.7
Ohio	6.2	(4.8–8.1)	7.6	(6.2–9.2)	6.4	(5.1–8.0)	3.2
Oklahoma	8.8	(6.8–11.2)	8.7	(6.9–10.8)	7.6	(6.0–9.6)	−13.6
Oregon	7.4	(5.3–10.1)	5.5	(3.5–8.4)	8.7	(6.4–11.6)	17.6
Pennsylvania	7.4	(6.0–9.3)	6.6	(5.3–8.1)	7.4	(6.0–9.1)	0.0
Rhode Island	4.3	(2.9–6.4)	2.0	(1.1–3.6)	5.5	(3.4–8.8)	27.9
South Carolina	5.6	(4.3–7.3)	5.5	(4.2–7.3)	6.3	(4.8–8.2)	12.5
South Dakota	10.0	(7.1–13.9)	7.8	(6.1–9.9)	9.1	(6.6–12.3)	−9.0
Tennessee	8.1	(4.8–13.2)	6.2	(4.6–8.4)	5.8	(4.0–8.4)	−28.4
Texas	8.8	(6.9–11.2)	7.5	(5.8–9.6)	8.2	(6.5–10.4)	−6.8
Utah	12.5	(10.2–15.2)	10.3	(8.0–13.2)	10.2	(8.1–12.9)	−18.4
Vermont	5.7	(4.0–8.2)	5.4	(3.7–7.7)	3.1	(2.0–4.8)	−45.6
Virginia	6.8	(4.8–9.2)	7.7	(5.5–10.7)	7.1	(5.5–9.1)	4.4
Washington	6.9	(5.1–9.2)	8.6	(7.0–10.5)	9.0	(7.1–11.2)	30.4
West Virginia	6.4	(4.8–8.5)	7.9	(6.2–9.9)	8.4	(6.7–10.6)	31.3¶
Wisconsin	8.2	(6.0–10.9)	7.9	(5.7–10.9)	8.3	(6.1–11.2)	1.2
Wyoming	11.6	(9.4–14.2)	12.1	(8.9–16.2)	12.8	(9.8–16.5)	10.3
**Median prevalence all states** **	**7.1**		**6.6**		**7.4**		**4.2**

**Abbreviations:** CI = confidence interval; RPC = relative percent change (see below).

* Persons aged ≥18 years who reported having smoked ≥100 cigarettes during their lifetime and smoke every day or some days at the time of survey.

† Persons aged ≥18 years who reported currently using chewing tobacco, snuff, or snus (a small pouch of smokeless tobacco) every day or some days at the time of survey.

§ RPC was calculated by dividing the difference between the 2013 and 2011 estimates by the 2011 estimates, and expressed as a percentage.

¶ p<0.05 for trend (2011–2013) in multivariate logistic regression model adjusted for sex, age, and race/ethnicity.

** Median prevalence across all 50 U.S. states and the District of Columbia.

## Footnotes

* Current cigarette smoking was assessed by two questions, “Have you smoked at least 100 cigarettes in your entire life?” and “Do you now smoke cigarettes every day, some days, or not at all?” Respondents who reported smoking at least 100 cigarettes in their lifetime and who reported currently smoking “every day” or “some days” were classified as current cigarette smokers.

† Respondents were classified as current smokeless tobacco users if they answered “every day” or “some days” to the following question, “Do you currently use chewing tobacco, snuff, or snus every day, some days, or not at all?” Snus (rhymes with “goose”) is a Swedish word for snuff and refers to a moist smokeless tobacco that is usually sold in small pouches that are placed under the lip against the gum.

§ Additional information available at http://www.cdc.gov/brfss/annual_data/2013/pdf/Weighting_Data.pdf.

¶ The RPC was calculated by dividing the difference between the 2013 and 2011 estimates by the 2011 estimates, and expressed as a percentage.

** Additional information available at http://www.cdc.gov/mmwr/preview/mmwrhtml/mm6122a3.htm.

†† Additional information available at http://www.tobaccofreekids.org/content/what_we_do/state_local_issues/settlement/FY2015/2014_12_11_brokenpromises_report.pdf.
